# Inter-correlations Among Clinical, Metabolic, and Biochemical Parameters and Their Predictive Value in Healthy and Overtrained Male Athletes: The EROS-CORRELATIONS Study

**DOI:** 10.3389/fendo.2019.00858

**Published:** 2019-12-10

**Authors:** Flavio A. Cadegiani, Claudio E. Kater

**Affiliations:** Adrenal and Hypertension Unit, Division of Endocrinology and Metabolism, Department of Medicine, Federal University of São Paulo Medical School, São Paulo, Brazil

**Keywords:** hormonal conditioning, endocrinology of physical activity, sports endocrinology, hormones and sports, endocrine and metabolic responses on overtraining syndrome (EROS) study, overtraining syndrome

## Abstract

**Objectives:** The Endocrine and Metabolic Responses on Overtraining Syndrome (EROS) study identified multiple hormonal and metabolic conditioning processes in athletes, and underlying mechanisms and biomarkers of overtraining syndrome (OTS). The present study's objective was to reveal independent predictors and linear correlations among the parameters evaluated in the EROS study to predict clinical, metabolic, and biochemical behaviors in healthy and OTS-affected male athletes.

**Methods:** We used multivariate linear regression and linear correlation to analyze possible combinations of the 38 parameters evaluated in the EROS study that revealed significant differences between healthy and OTS-affected athletes.

**Results:** The testosterone-to-estradiol (T:E) ratio predicted the measured-to-predicted basal metabolic rate (BMR) ratio; the T:E ratio and total testosterone level were inversely predicted by fat mass and estradiol was not predicted by any of the non-modifiable parameters. Early and late growth hormone, cortisol, and prolactin responses to an insulin tolerance test (ITT) were strongly correlated. Hormonal responses to the ITT were positively correlated with fat oxidation, predicted-to-measured BMR ratio, muscle mass, and vigor, and inversely correlated with fat mass and fatigue. Salivary cortisol 30 min after awakening and the T:E ratio were inversely correlated with fatigue. Tension was inversely correlated with libido and directly correlated with body fat. The predicted-to-measured BMR ratio was correlated with muscle mass and body water, while fat oxidation was directly correlated with muscle mass and inversely correlated with fat mass. Muscle mass was directly correlated with body water, and extracellular water was directly correlated with body fat and inversely correlated with body water and muscle mass.

**Conclusions:** Hypothalamic-pituitary responses to stimulation were diffuse and indistinguishable between the different axes. A late hormonal response to stimulation, increased cortisol after awakening, and the T:E ratio were correlated with vigor and fatigue. The T:E ratio was also correlated with body metabolism and composition, testosterone was predicted by fat mass, and estradiol predicted anger. Hydration status was inversely correlated with edema, and inter-correlations were found among fat oxidation, hydration, and body fat.

## Introduction

The benefits of extensive exercise have exceeded previous expectations, including primary prevention, active part of the treatment, improvement of life quality, and prognosis under chronic or incurable diseases, and prevention of complications and recurrence, of a wide range of diseases, including cardiovascular (1, 2), hypertension, type 2 diabetes mellitus (T2DM) (1), dyslipidemia, cancers (3–6), cognitive function (7), and all quality of life domains (8–10).

Many of the benefits from physical activity are linked to multiple adaptive changes that leads to improvements in neuromuscular (11), cardiovascular (12), musculoskeletal, autonomic, and other systems that active individuals undergo. However, these benefits may only occur when physically active individuals sleep and eat appropriately, alongside with training (13, 14).

Indeed, overtraining syndrome (OTS), which affects between 40 and 60% of the elite athletes during their careers, is likely a major manifestation of the harms of an imbalance between excessive training, insufficient recovery, non-refreshing sleep, insufficient caloric, protein, and/or carbohydrate intake, and concurrent psychological stress, including excessive cognitive effort, social, familiar, or financial issues (15–17). This imbalance is the likely underlying reason of the paradoxical loss of physical performance in OTS, which is not able to be justified by any apparent dysfunction (15). However, OTS is still a controversial issue since several characteristics of OTS, including underlying mechanisms, pathophysiology, and biological markers are universally accepted or clarified, as the prevailing findings on previous studies were inconsistent (15–17).

To address all the unanswered questions on OTS, and also to better understand the multiple conditioning processes that athletes seem to undergo, we conducted the Endocrine and Metabolic Responses to Overtraining Syndrome (EROS) study (18–24). In that study, we evaluated basal and exercise-independent hormonal responses to stimulation tests, multiple biochemical markers, including muscular, inflammatory, immunologic, and nutritional parameters, specific eating, psychological, and social patterns, and the body metabolism and composition of healthy athletes, athletes affected by OTS, and sedentary individuals with similar baseline characteristics (age, sex, and body mass index—BMI). The list of parameters evaluated by the EROS study are detailed in Table 1, together with the selection process for the present analysis, to be described further.

**Table 1:** Eligible markers for the present analysis.

**Tests**	**Markers**	**Initially elected? (Provide independent data, provide additional data (in relation to other parameters evaluated), substantiated/validated)**	**Significantly different between OTS-affected and healthy athletes? (If “Yes,” included in the present analysis)**
**BASAL BIOCHEMICAL TESTS**			
Basal hormones	(1) Total testosterone (ng/dL) (2) Estradiol (pg/mL) (3) IGF-1 (pg/mL) (4) TSH (μUI/mL) (5) Free T3 (pg/mL) (6) Total catecholamines (μg/12 h) (7) Total metanephrines (μg/12 h) (8) Noradrenaline (μg/12 h) (9) Epinephrine (μg/12 h) (10) Dopamine (μg/12 h) (11) Metanephrines (μg/12 h) (12) Normetanephrines (μg/12 h)	Yes Yes Yes Yes Yes Yes Yes Yes Yes Yes Yes Yes	**Yes** **Yes** No No No **Yes** No No No **Yes** No No
Muscular, inflammatory, immunologic, and other basal biochemical markers	(13) Erythrocyte sedimentation rate (ESR, mm/h) (14) Hematocrit (%) (15) C-reactive protein (CRP, mg/dL) (16) Lactate (nMol/L) (17) Vitamin B12 (pg/mL) (18) Ferritin (ng/mL) (19) Neutrophils (mm^3^) (20) Lymphocyte (mm^3^) (21) Eosinophils (mm^3^) (22) Creatine kinase (CK, U/L) (23) Low-density lipoprotein cholesterol (LDLc, mg/dL) (24) High-density lipoprotein cholesterol (HDLc, mg/dL) (25) Tryglycerides (mg/dL) (26) Medium corpuscular volume (MCV) (27) Platelets (10^3^/mm)	Yes Yes Yes Yes Yes Yes Yes Yes Yes Yes Yes Yes Yes No (interpretation may vary) No (platelet-to-lymphocyte was used instead)	No No No **Yes** No No **Yes** Yes^*^ No **Yes** No No No - -
Ratios	(28) Testosterone-to-oestradiol ratio (29) Testosterone-to-cortisol ratio (30) Neutrophil-to-lymphocyte ratio (31) Platelet-to-lymphocyte ratio (32) Catecholamine-to- metanephrine ratio	Yes Yes Yes Yes No (Non-validated marker)	**Yes** No **Yes** Yes^*^ -
**HORMONAL FUNCTIONAL TESTS**			
Insulin tolerance test (ITT)	(33) Basal ACTH (pg/mL) (34) ACTH during hypoglycaemia (pg/mL) (35) ACTH 30 min after hypoglycemia (pg/mL) (36) ACTH increase during ITT (pg/mL) (37) Basal cortisol (μg/dL) (38) Cortisol during hypoglycaemia (μg/dL) (39) Cortisol 30 min after hypoglycemia (μg/dL) (40) Cortisol increase during ITT (μg/dL) (41) Basal GH (μg/L) (42) GH during hypoglycaemia (μg/L) (43) GH 30 min after hypoglycemia (μg/L) (44) Basal prolactin (ng/mL) (45) Prolactin during hypoglycaemia (ng/mL) (46) Prolactin 30 min after hypoglycemia (ng/mL)	Yes Yes Yes Yes Yes Yes Yes Yes Yes Yes Yes Yes Yes Yes	No No **Yes** **Yes** No **Yes** **Yes** No **Yes** **Yes** **Yes** **Yes** **Yes** **Yes**
	(47) Prolactin change during ITT (ng/mL) (48) Basal ACTH/cortisol ratio (49) ACTH/cortisol ratio during hypoglycemia (50) ACTH/cortisol ratio 30 min after hypoglycaemia (51) Basal serum glucose (mg/dL) (52) Serum glucose during hypoglycemia (mg/dL) (53) Capillary glucose during hypoglycemia (mg/dL) (54) Adrenergic symptoms during hypoglicemia (0–10) (55) Neuroglycopenic symptoms during hypoglicemia (0–10)	Yes No (Non-validated marker) No (Non-validated marker) No (Non-validated marker) No (does not provide useful data) No (does not provide useful data) No (does not provide useful data) No (Non-validated marker) No (Non-validated marker)	No - - - - - - - -
*Cosyntropin* stimulation test (CST)	(56) Basal cortisol (μg/dL) (57) Cortisol at 30 min after infusion (μg/dL) (58) Cortisol at 60 min after infusion (μg/dL) (59) Difference between basal cortisol on day 1 (CST) and day 3 (ITT) (%)	No (does not provide additional data) Yes Yes No (non-validated marker)	- No No -
Salivary cortisol rhythm (SCR**)**	(60) Salivary cortisol (ng/dL) at awakening (61) Salivary cortisol (ng/dL) 30 min after awakening (62) Salivary cortisol (ng/dL) at 4 p.m. (63) Salivary cortisol (ng/dL) at 11 p.m. (64) Cortisol awakening response (CAR) (65) Difference between 8 a.m. and 4 p.m. salivary cortisol (%)	Yes Yes Yes Yes Yes No (non-validated marker)	No **Yes** No No No -
**CLINICAL PARAMETERS**			
Sleeping and social characteristics	(66) Duration of night sleep (h/night) (67) Self-reported sleep quality (0–10) (68) Self-reported libido (0–10) (69) Number of hours of activities besides professional training (h/day) (70) Initial imnsonia (Y/N) (71) Terminal imnsonia (Y/N) (72) More than two wake-ups during sleep (Y/N) (73) Work and/or study (Y/N) (74) Libido during resting periods / vacations (0–10)	Yes No (out of the scope of the present study) Yes Yes No (qualitative marker) No (qualitative marker) No (qualitative marker) No (qualitative marker) No (qualitative marker)	No No **Yes** No - - - - -
	(75) Calorie intake (kcal/kg/day) (76) Carbohydrate intake (g/kg/day) (77) % calories from carbohydrate (%) (78) Protein intake (g/kg/day) (79) % calories from protein (%) (80) Fat intake (g/kg/day) (81) % calories from fat (%) (82) Carbohydrate intake > 3 g/kg/day (Y/N) (83) Daily whey protein consumption (Y/N) (84) Followed a diet plan (Y/N)	No (out of the scope of the present study) No (out of the scope of the present study) No (out of the scope of the present study, and intrinsically linked to other markers) No (out of the scope of the present study) No (out of the scope of the present study, and intrinsically linked to other markers) No (out of the scope of the present study) No (out of the scope of the present study, and intrinsically linked to other markers) No (out of the scope of the present study and a qualitative marker) No (out of the scope of the present study and a qualitative marker) No (out of the scope of the present study and a qualitative marker)	*-* *-* *-* *-* *-* - - - - -
	(85) Post-workout carbohydrate intake > 0.5 g/kg (Y/N; only applicable for athletes)	No (out of the scope of the present study and a qualitative marker)	-
Psychological patterns	(86) Profile of Mood State (POMS) scale (total score:−32 to +120) (87) Anger subscale (0–48) (88) Confusion subscale (0–28) (89) Depression subscale (0–60) (90) Vigor subscale (0–32) (91) Fatigue subscale (0–28) (92) Tension subscale (0–36) (93) Have you been sick in the last 2 weeks? (Y/N) ? (94) How was your last training session compared to the projected goals? (Extremely easy to extremely hard) (95) How do your muscles feel? (Nothing at all to extremely painful) (96) How friendly do you feel today? (0–6) (97) How worthless do you feel today? (0–6) (98) How miserable do you feel today? (0–6) (99) How helpful do you feel today? (0–6) (100) How bad-tempered do you feel today? (0–6) (101) How unworthy do you feel today? (0–6) (102) How peeved do you feel today? (0–6) (103) How cheerful do you feel today? (0–6) (104) How sad do you feel today? (0–6) (105) How do you fell today? (0–10)	Yes Yes Yes Yes Yes Yes **Yes** No (alone does not determine status, and a qualitative marker) No (alone does not determine status) No (alone does not determine status) No (alone does not determine status) No (alone does not determine status) No (alone does not determine status) No (alone does not determine status) No (alone does not determine status) No (alone does not determine status) No (alone does not determine status) No (alone does not determine status) No (alone does not determine status) No (alone does not determine status)	**Yes** **Yes** **Yes** **Yes** **Yes** **Yes** **Yes** - - - - - - - - - - - - -
Body metabolism analysis	(106) Measured-to-predicted basal metabolic rate (BMR, %) (107) Percentage of fat burning compared to total BMR (%)	Yes **Yes**	Yes **Yes**
Body composition	(108) Body fat percentage (%) (109) Muscle mass percentage (%) (110) Body water percentage (BW, %) (111) Extracellular water compared to total BW (%) (112) Visceral fat (cm^2^) (113) Chest-to-waist circumference ratio (114) Waist circumference (cm) (115) Chest circumference (cm) (116) Biceps circumference (cm) (117) Hip circumference (cm)	Yes Yes Yes Yes Yes Yes No (interpretation may vary, and alone does not determine status or diagnosis) No (interpretation may vary, and alone does not determine status or diagnosis) No (interpretation may vary, and alone does not determine status or diagnosis) No (interpretation may vary, and alone does not determine status or diagnosis)	**Yes** **Yes** **Yes** Yes^*^ **Yes** Yes^*^ - - - -

* *p > 0.05 but < 0.1 between OTS-affected and healthy athletes, but different between athletes (both groups) and sedentary, with possible clinical significance. Bold values: parameters that were selected for the present analysis*.

**Figure 1:**
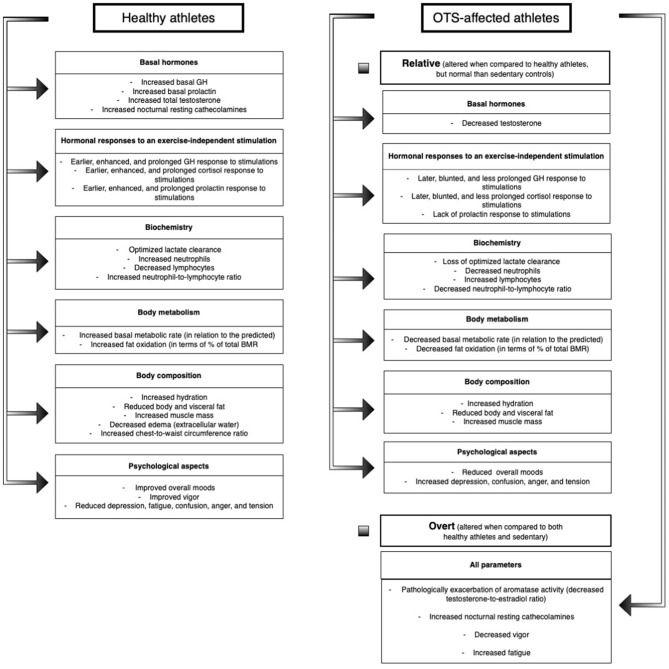
Main findings of the EROS study.

Among the 117 markers evaluated in the EROS study, we identified 50 novel parameters for OTS-affected and healthy athletes, including amplified and prolonged GH, cortisol, and prolactin responses to a stimulation test, increased testosterone, lactate clearance, catecholamines, basal metabolic rate (BMR), fat oxidation, and hydration in healthy athletes, and blunted hormonal responses (compared to healthy athletes), increased creatine kinase (CK), aromatase activity, estradiol, anger, depression, fatigue, mental confusion and fat mass, and reduced testosterone, hydration, muscle mass, BMR, fat oxidation, and moods in athletes affected by OTS (23). The major findings of the EROS study are described in Figure 1.

The findings unveiled by the EROS study supported the hypothesis of the existence of multiple adaptations of clinical, metabolic, biochemical, and body parameters that athletes, while the majority of the physiological adaptive changes are compromised in OTS, which may explain the hallmark of OTS, the loss of performance (23, 24).

Despite the multiple and broad adaptive changes previously demonstrated to occur in athletes, and the more than 50 novel markers and processes identified in both healthy and OTS-affected athletes in the present study, the relationships between parameters that are affected by training and/or OTS are unclear (18–21). Associations, interactions, synergisms, stimulations, and inhibitions between hormones, inflammatory, immunologic, muscular, metabolic, and clinical markers, and psychological, eating, sleep, and training patterns, have been poorly assessed previously, and have not been identified in the EROS study, once our primary objective was to detect differences between OTS and healthy athletes, and sedentary control among the 117 parameters evaluated, using three-group and pairwise comparisons, which were published in different arms (18–24).

The unexpected large number of markers identified in both populations of athletes allowed us hypothesize the existence of a web of multiple sorts of interactions between parameters of different natures, which could result in the wide range of benefits and improved performance observed in healthy athletes, and the paradoxical decrease of sports performance, fatigue, reduced libido, and body changes in OTS (24). The correlations to be identified between the newly uncovered parameters could provide a new understanding of the complex processes of conditioning processes that athletes typically undergo, and the convoluted mechanisms that lead to OTS (23, 24).

In summary, despite the large number of discoveries (18–22, 24), our primary findings do not demonstrate the multiple sorts of relationship between those markers that participate in the adaptative processes of the athletes and those that have roles in the pathogenesis of OTS. Therefore, in the present study we aimed to uncover the web of multiple interactions that participate in the conditioning processes that occur in athletes, and the underlying mechanisms of the pathophysiology of OTS, derived from an exhaustive yet reasonable joint *post-hoc* analysis of the primary findings of the EROS study, using different and more complex statistical analyses (e.g., multivariate linear regression, logistic regression, and linear correlation analyses) that those employed in the primary arms of the study, which is adequate owing to the large number of data generated by the EROS study (more than 11,000 results among 117 parameters).

Our ultimate objective was to identify independent predictors and linear correlations, and determine causal relationships and inter-influences, among hormonal, immunologic, inflammatory, muscular, psychological, metabolic, and body composition parameters, aiming to uncover behavior patterns and dysfunctional pathways in OTS.

## Materials and Methods

### Subject and Parameter Selection

The detailed methodology was of the EROS study was previously published (18–22), also available at a depository (https://osf.io/bhpq9/). The present study was approved by the ethical committee of the Federal University of São Paulo (Approval Number: 1093965).

**Figure 2:**
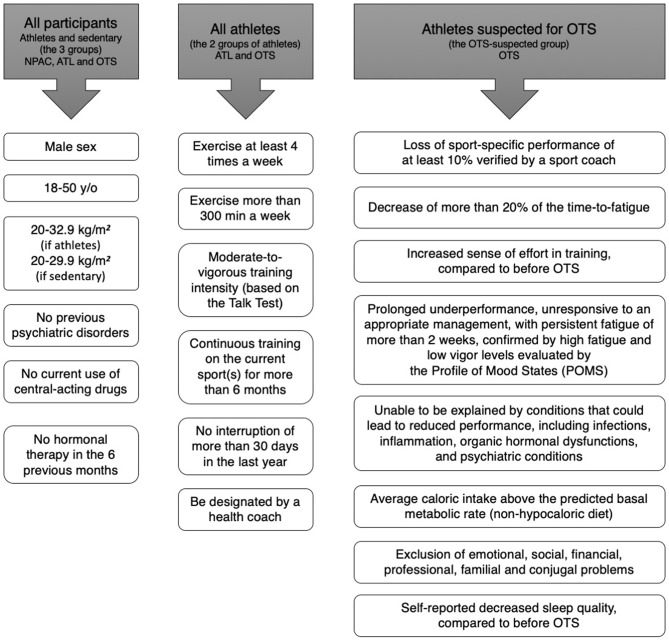
Selection criteria for the EROS study.

We recruited participants through social media (Facebook, Instagram, Whatsapp), and were initially evaluated for age, sex, weight and height, clinical characteristics, and if they were suspected for Overtraining Syndrome (OTS), healthy athletes (ATL), or non-physically active (NPAC), and training (if athletes). Inclusion criteria for all participants, criteria for all athletes, and specific criteria for OTS are shown in Figure 2. Employing a two-step selection process, we avoided athletes with an insufficient amount of training for the adaptive changes to exercises, non-full sedentary, extremes of age, misdiagnosis of OTS, and presence of confounding hormones, medications, and diseases.

For OTS candidates, we employed the diagnostic flowchart proposed by the latest guideline on OTS (15), the joint guideline of the European College of Sport Science and American College of Sports Medicine, from 2013, which requires the presence of decrease of at least 10% in training intensity, volume, pace, power, strength, or overall performance, decrease of the time-to-fatigue of at least 20%, both verified by a professional coach, increased sense of effort, changes in behavior and decreased energy levels, with or without sleep disturbances, infections or injuries, which persisted for at least 1–2 months, despite the attempts to recover, and which is not caused by conditions that could inherently lead to decrease of performance, including inflammations, infections, and frank hormonal dysfunctions.

#### Design of the Study

##### Summary of the procedures according to each primary arm of the EROS study

All selected participants signed a written informed consent for participation in the study, approved by the ethics committee of the Federal University of São Paulo, in accordance with the Declaration of Helsinki. Then, participants underwent hormonal responses to stimulation tests, basal biochemical, inflammatory, muscular, immunologic, and hormonal parameters, nocturnal urinary catecholamines (NUC) and its metabolites, analysis of body metabolism and composition, and evaluation of psychological, social, sleep, and eating patterns.

In the EROS-HPA axis arm of the study, we evaluated peripheral and central components of the hypothalamic-pituitary-adrenal (HPA) axis (whether primary or peripheral: adrenal, or central: pituitary and hypothalamus), by employing a 250 ug cosyntropin stimulation test (CST), for direct evaluation of cortico-adrenal responses to a synthetic ACTH, an insulin tolerance test (ITT), an exercise-independent stimulation test that provokes a hypoglycemia as the stimulation for the evaluation of the integrity of the HPA axis, and salivary cortisol rhythm (SCR), for the identification of the patterns of the circadian rhythm of the cortisol.

In the EROS-STRESS test we employed the same ITT for the evaluation of the of the growth hormone (GH) and prolactin responses to hypoglycemia, and which we detailed different aspects of the test, including time-to-hypoglycemia, glycemic nadir, severity of adrenergic, and neuroglycopenic symptoms during hypoglycemia, and compared between groups.

In the EROS-BASAL arm, we evaluated basal hormones, immunologic, muscular, classical inflammatory, lipids, and vitamin. And for the EROS-PROFILE, participants underwent several questions regarding specific sleeping, eating, social, and psychological patterns, and underwent analysis of body composition and metabolism.

The full process was performed during a short period, of <10 days, between the recruitment, clinical, and biochemical inclusion and exclusion criteria, the collect of the basal biochemical parameters, all questionnaires, body composition, and metabolism, and functional tests. For all parameters we performed three-group and pairwise comparisons.

### Procedures and Tests

#### Questionnaires

After the selection criteria, athletes (sedentary subjects were not assessed at this moment) underwent an initial specific interview about training patterns, including the type(s) the sport(s) practiced, time since starting the current sport(s), training volume and intensity (evaluated by a professional coach, on a scale from 0 to 10 compared to athletes of the same level of training), duration of training per week (min), number of rest days per week was recorded based on standardized tests, and whether they were supervised by a coach. This first questionnaire aimed to determine the baseline characteristics of the OTS and ATL Groups.

For all athletes affected by OTS, we evaluated whether and the number of days to overcome the underperformance state, changes in sensitivity to heat or to coldness, occurrence of infections, particularly upper respiratory tract infections (URTIs), and injuries, and feelings of monotony and boredom.

All participants (now including the non-physically active group—NPAC) then underwent specific questionnaires regarding sleeping, eating, social, and psychological characteristics.

In terms to eating habits, we employed a 7-day food and nutrition specific record and manually calculated mean daily carbohydrate, fat, protein, and overall caloric intake, aiming to preclude heterogeneity regarding the food analysis. The following specific aspects were evaluated: mean daily (1) carbohydrate, (2) protein, and (3) fat intake (in g/kg), (4) mean daily calorie, total (kcal/day) and per weight (kcal/kg/day), (5) the proportion of carbohydrate, protein and fat intake, (6) whether a diet plan was followed (yes or no), (7) whether there was daily whey protein ingestion, (8) whether post-training carbohydrate intake was > 0.5 g/kg, and (9) whether carbohydrate intake throughout the day was > 4.0 g/kg.

With regards to sleeping patterns, evaluation included the following aspects: (1) self-reported sleep quality (zero to ten; where zero = awful and ten = great), (2) mean duration of sleeping time, (3) whether there was difficulty falling sleep, (4) whether waking up too early and unable to sleep again, and (5) whether waking up more than two times during the night.

For the evaluation of the social aspects, we asked (1) whether participants attended work or study besides the professional training sessions, if so, (2) how many hours per day they attended to the professional activities besides the training periods, and (3) their self-reported libido, from zero to ten (zero = no sex drive; ten = astonishing sex drive), compared to 1 year before, during the training periods.

With regards to the psychological characteristics, we employed the Profile of Mood States (POMS) questionnaire with the overall and specific mood scales: (1) total score (from −32 to +200; where −32 is the best score and +200 the worst score), (2) anger (from 0 to 48), (3) confusion (from 0 to 28), (4) depression (from 0 to 60), (45 fatigue (from 0 to 28), (6) tension (from 0 to 36), and (7) vigor (from 0 to 32; vigor score is counted as negative for the total POMS score) subscales. In a different moment of the interview, we also evaluated specific self-reported feelings of (8) general well-being, (9) friendly, (10) worthless, (11) miserable, (12) helpful, (13) bad-tempered, (14) guilty, (15) unworthy, (16) peeved, (17) cheerful, (18) sad, and (19) fatigue [from zero (not fatigued at all) to ten (extremely fatigued)].

The POMS questionnaire and the specific feeling questions were performed by only one author (FAC), in an impartial way, with a constant voice and standardized words of each question in order to prevent “faking good” in ATL and “faking bad” in healthy and OTS-affected athletes, respectively. The RESTQ questionnaire, also used to evaluate athletes, although not validated for non-physically active individual, was not employed, once NPAC were also evaluated as a second group control.

#### Basal Tests

Between 36 and 48 h after the last training session (in the case of the groups of athletes), we collected basal fasting levels of the following parameters: CRP; ESR; creatinine; hematocrit, medium corpuscular volume, and numbers of neutrophils, lymphocytes, eosinophils, and platelets (automated assays); CK; ferritin; high-density lipoprotein-cholesterol and triglycerides (calorimetric enzymatic assays) and low-density lipoprotein-cholesterol (Friedewald equation); **serum** lactate (enzymatic assays); serum total testosterone; estradiol (chemiluminescence assay); serum IGF-1 (chemiluminescence assay); nocturnal 12-h urinary catecholamines and metanephrines (calorimetric enzymatic assays); serum free thyronine (fT3; electrochemiluminescence assay); and serum TSH (electrochemiluminescence assay).

We then calculated the testosterone-to-estradiol, testosterone-to-cortisol, catecholamines-to-metanephrines, neutrophil-to-lymphocyte, and platelet-to-lymphocyte ratios, and compared them between the groups.

#### Hormonal Functional Tests

The CST, ITT, and SCR were performed in all participants, in a specific sequence.

##### Cosyntropin stimulation test (CST)

In the first day, we performed a stimulation test with a high doses (250 μg) of cosyntropin, a synthetic adrenocorticotropic hormone (ACTH), in order to hormonal responsiveness of the adrenal glands.

For the CST, at 8.00 a.m. (after 30-min resting and 8-h fasting) blood was collected (time 0) from the antecubital vein of the participants for serum cortisol. Immediately, 250 μg of cosyntropin was infused intravenously, slowly (during 30 s), and blood was collected at 30 min (time 1) and 60 min (time 2) for the analysis of the cortisol increase, in absolute levels [μg/dL], in response to a synthetic ACTH.

##### Insulin tolerance test (ITT)

Forty-eight hours after the CST, we then performed an ITT, to evaluate the integrity of the HPA, GH, and prolactin axes, once a normal response required absolute unaltered functions in all levels (hypothalamus, pituitary, and adrenals or other glands) of the axes. This is an intrinsic and independent test of the hormonal responsiveness, without interferences from external signaling or systems.

Participants followed the same protocol of at least 8-h fasting, arrival time before 7.30 a.m. and a 30- resting period prior to the beginning of the ITT. Although participants had signed the written consent and were fully aware of the risks of an ITT, before the beginning of the ITT we reminded them of the potential side effects derived from a state of hypoglycemia purposely induced by the test. After agreeing, blood was collected (time 0), and a dose of 0.1 IU/kg of regular insulin was infused in bolus. When hypoglycemia was detected, blood was collected (time 1—during hypoglycemia), 10 mL of 50% glucose solution was given intravenously, and high-glycemic index food was offered *ad libitum* (fat free ice-creams, Diletto, São Paulo, Brazil), blood was finally sampled again, 30 min (time 2) and 60 min (time 3) after the hypoglycemic episode.

The criteria for the detection of the hypoglycemia for the collect of the blood at time 1 was: (1) Asymptomatic hypoglycemia, when capillary glucose was below 30 mg/dL; (2) Moderate-to-intense adrenergic (cold sweating, shakiness, pallor, heart palpitations) and/or neuroglycopenic (mood changes, unrest, sleepiness) symptoms (a score of 5–10, from a zero to ten scale), regardless of the glucose levels; and (3) Capillary glucose below 45 mg/dL associated with any adrenergic or neuroglycopenic symptom.

Serum glucose (mg/dL), cortisol (μg/dL), ACTH (pg/mL), GH (μg/L), and prolactin (ng/mL) were collected at all times. During the ITT, time-to-hypoglycemia (minutes since the insulin infusion), and level of intensity of adrenergic and neuroglycopenic symptoms (zero to ten, self-reported) were also evaluated during the ITT. Absolute increase of cortisol, prolactin, GH, and ACTH, as well as the ACTH/cortisol ratio at all times were calculated. Among these hormones, we adjusted GH for body composition, since GH release is negatively influenced by body fat.

Given the actual risk of ITT-induced severe hypoglycemia (loss of consciousness), three doses of subcutaneous glucagon (GlucaGen HypoKit, 1 μg, NovoNordisk), syringes containing 20 mL of 50% glucose solution and an automated external defibrillator (AED) were available.

##### Salivary cortisol rhythm (SCR)

Between 2 and 7 days after the ITT, we collected the SCR, including the collect of the saliva at the awakening moment, at 30 min after awakening, at 4 and at 11 p.m. which were collected by the participants themselves, using laboratory kits provided by the researcher (FAC). Specific recommendations for the self-collect of the samples were provided.

All hormones from the functional tests were analyzed by specific electrochemiluminescence assays using specific commercial kits (Roche), while serum glucose was analyzed by an enzymatic assay of hexokinase.

All biochemical data were determined using standardized commercial assay kits ((18–21), https://osf.io/bhpq9). The inter- and intra-assay coefficients of variability were lower than 3.5 and 3%, respectively.

#### Body Composition and Metabolism

On a different day from the CST, ITT, or CST, previously scheduled, and after at least 24 h of the last training session (for OTS and ATL), we performed the evaluation of body composition using a gold-standard air-displacement pletimosgraph (Bod Pod, CosMed, USA) for analysis of body fat in terms of weight (kg) and percentage (%), and a validated and standardized electrical bioimpedance scale (InBody770, Biospace, South Korea) for analysis of visceral fat (%), muscle mass (kg), percentage of lean mass (%), body water (liters), percentage of body water (%), and percentage of extracellular water (%).

We then measured chest, biceps, and waist circumferences using a standardized and highly accurate pro-body- scanner and (Styku, USA). An indirect calorimetry (Spirostik, Geratherm Respiratory, Germany) was performed to evaluate basal metabolic rate (BMR) (kcal/day), the measured-to-predicted BMR ratio (%), after adjustments for age, weight, height and sex, and fat oxidation in relation to total metabolic rate (%).

#### Selection of the Markers for the Analysis of Associations, Predictions, and Correlations Between Markers

We initially excluded 48 of the 117 markers present in the EROS study, by excluding those that were intrinsically linked to other parameters (seven markers), did not determine diagnoses or status (16 markers), were qualitative indices (nine markers), markers that did not provide additional independent data (two markers), markers that were not the behavioral consequences of exercise (four markers), and invalid and/or unsubstantiated data (nine markers) (Table 1). We then selected the 38 from the 69 remaining markers that yielded significant differences between the two groups of athletes: OTS-affected athletes and healthy athletes (34 markers), or that were significantly different between these groups and sedentary controls (*p* < 0.05) with trends to be significantly different between healthy and OTS-affected athletes (*p* < 0.1) (four markers).

### Statistical Analysis

We used multivariate linear regression to analyze all possible combinations of the 38 parameters that were evaluated in the EROS study (18–21). The intent was to identify: (1) clinical or biochemical markers (including hormonal, metabolic, and body metabolism and composition markers) as independent predictors of other markers and (2) strong linear correlations among the parameters evaluated in the EROS study (18–21). As the identification of triggers and the influence of OTS were not evaluated in the present study, they were excluded from the analyses.

We used multivariate linear regression with a removal criterion of *p* > 0.01. The standardized residual variables of the last model were examined for normality and homoscedasticity. The cut-off for the presence of multicollinearity was a tolerance index of 0.40^3^ for the variables in the last model. A *p*-value < 0.05 was considered statistically significant for independent predictors, and *p* < 0.01 for linear correlations (with >0.40 correlation coefficient), which we considered to unveil moderate-to-strong correlations.

Although *r* > 0.4 is generally considered to be of moderate association, there is no rule or universally accepted sizes of correlation to be considered as weak, moderate or strong. Since we studied entirely different biological aspects, and each of these aspects is extensively influenced by a large number of different predictors from different natures, it is unlikely to find a single linear correlation > 0.5 (> −0.5), since each parameter tends to be driven by multiple factors. Hence, in this particular case, according to the literature, a correlation > 0.4 is sufficient to be considered as a strong correlation (25–27), or at least moderate-to-strong.

The *p*-value for the linear correlations was lower and partial correlations were not considered to avoid incidental misinterpretative correlations. Analyses were performed using SAS 9.4 (SAS Institute, Inc., Cary, NC, USA).

Parameters that were independently influenced by OTS, as published in the EROS-DISRUPTORS study (24), were adjusted according to the level of influence of OTS, in order to homogenize the groups of athletes. The biological behaviors that were modified by the presence of OTS include: (1) cortisol 30 min after hypoglycemia, in response to an ITT (26.1% of influence by OTS); (2) cortisol increase during ITT (22.0%); (3) GH 30 min after hypoglycemia, in response to an ITT (23.0%); (4) testosterone-to-estradiol (T:E) ratio (30.7%); (5) neutrophils (13.8%); (6) neutrophil-to-lymphocyte ratio (13.6%) (7) Profile of Mood States (POMS) vigor subscale (83.6%); (8) POMS fatigue subscale (85.7%); (9) POMS tension subscale (42.8%); (10) muscle mass (33.7%); (11) body water (50.5%), and (12) visceral fat (38.2%).

Those parameters that were not modified by the presence of OTS did not require adjustments according to the population (if OTS-affected or if healthy athletes), since these markers behaved independently from OTS.

Correlations that were unlikely to have any biological plausibility were excluded.

Further information on the material, methods, individualized results, and the raw data are provided at a repository (https://osf.io/bhpq9/).

## Results

### Primary Results

All sub-groups had similar age, BMI, and training patterns. As per selection criteria, all 34 parameters were significantly different between OTS-affected and healthy athletes. The primary results of these markers are detailed in Table 2.

**Table 2:** Primary results of the markers selected for the present analysis.

**Tests**	**OTS-affected athletes**	**Healthy athletes**	**Significance**
**BASAL BIOCHEMICAL TESTS**			
Total testosterone (ng/dL)	422 (±173.2)	540.3 (±171.4)	*p* = 0.008
Estradiol (pg/mL)	40.1 (±10.8)	29.8 (±13.9)	*p* = 0.001
Total catecholamines (μg/12 h)	257 (±66)	175 (±69)	*p* = 0.015
Dopamine (μg/12 h)	227 (±159)	149 (±60)	*p* = 0.01
Salivary cortisol (ng/dL) 30 min after awakening	324 (±116)	500(±168)	*p* = 0.005
Lactate (nMol/L)	1.11(0.79 to 2.13)	0.78 (0.47-1.42)	*p* = 0.003
Neutrophils (mm^3^)	2986 (±761)	3809 (±1431)	*p* = 0.022
Creatine kinase (CK, U/L)	569 (126 to 3012)	347 (92 to 780)	*p* = 0.038
Testosterone-to-oestradiol ratio	10.8 (±3.7)	20.8 (±9.9)	*p* <0.001
Neutrophil-to-lymphocyte ratio	1.23(±0.34)	2.00 (±1.28)	*p* = 0.008
Lymphocyte (mm^3^)^*^	2498 (±760)	2154 (±640)	^*^(NPAC = 2819 ± 810) *p* = 0.03 (overall), *p* = 0.018 (NPAC x ATL) and *p* = 0.224 (NPAC x OTS)
Platelet-to-lymphocyte ratio^*^	104.1 (±34.2)	119.1 (±43.4)	^*^(NPAC = 82.4 ± 19.5) *p* = 0.017 (overall), *p* = 0.003 (NPAC x ATL) and *p* = 0.102 (NPAC x OTS)
**HORMONAL FUNCTIONAL TESTS**			
Basal GH (μg/L)	0.1 (0.05 to 0.87)	0.26 (0.1 to 1.26)	*p* = 0.007
Basal prolactin (ng/mL)	9.2 (5.27 to 19.46)	12.1 (7.19 to 23.0)	*p* = 0.048
Cortisol during hypoglycaemia (μg/dL)	12.4 (±3.3)	15.9 (±5.3)	*p* = 0.022
GH during hypoglycaemia (μg/L)	0.4 (0.05 to 4.68)	2.5 (0.08 to 40.94)	*p* = 0.012
Prolactin during hypoglycaemia (ng/mL)	8.95 (4.72 to 47.22)	17.85 (10.0 to 63.39)	*p* <0.001
ACTH 30 min after hypoglycemia (pg/mL)	30.3 (9.8–93.7)	59.9 (22.1 to 195.7)	*p* <0.001
Cortisol 30 min after hypoglycemia (μg/dL)	17.9 (±2.9)	21.7 (±3.1)	*p* <0.001
GH 30 min after hypoglycemia (μg/L)	1.28 (0.03 to 13.95)	12.73 (1.1 to 38.1)	*p* <0.001
Prolactin 30 min after hypoglycemia (ng/mL)	11.35 (4.5 to 25.88)	24.3 (10.5 to 67.45)	*p* <0.001
ACTH response to ITT (pg/mL)	9.7 (−14.4 to +64.4)	45.1 (22.1 to 195.7)	*p* <0.001
**CLINICAL PARAMETERS**			
Self-reported libido (0–10)	6.2 (±2.1)	8.3 (±1.7)	*p* = 0.004
POMS questionnaire (Total score: −32 to +120)	+54.5 (−14.8 to +89.2)	−9.0 (−23.4 to +17.2)	*p* <0.001
Anger subscale (0 to 48)	15.0 (4.0 to 21.0)	5.0 (0.2 to 15.0)	*p* = 0.003
Confusion subscale (0 to 28)	5.0 (1.6 to 17.1)	2.00 (0.0 to 5.0)	*p* = 0.001
Depression subscale (0 to 60)	7.5 (0.0 to 21.4)	0 (0.0 to 5.0)	*p* = 0.008
Vigor subscale (0 to 32)	9.5 (3.6 to 20.1)	26.0 (21.2 to 28.0)	*p* <0.001
Fatigue subscale (0 to 28)	20.0 (9.3 to 26.7)	2.0 (0.0 to 4.8)	*p* <0.001
Tension subscale (0 to 36)	16.5 (3.6 to 20.1)	6.0 (1.0 to 14.4)	*p* <0.001
**BODY PARAMETERS**			
Measured-to-predicted basal metabolic rate (BMR, %)	102.6 (±8.3)	109.7 (±9.3)	*p* = 0.012
Percentage of fat burning compared to total BMR (%)	33.5 (±21.0)	58.7 (±18.7)	*p* <0.001
Body fat percentage (%)	17.0 (±6.0)	10.8 (±4.2)	*p* <0.001
Muscle mass percentage (%)	47.2 (±3.8)	50.5 (±2.3)	*p* = 0.008
Body water percentage (BW, %)	59.5 (±3.9)	64.7 (±2.7)	*p* <0.001
Visceral fat (cm^2^)	67.5 (±36.5)	35.7 (±20.6)	*p* = 0.01
Extracellular water compared to total BW (%)^*^	20.1 (±12.0)	21.8 (±11.8)	^*^(NPAC = 33.1 ± 16.7) *p* = 0.022 (overall), *p* = 0.019 (NPAC x ATL) and *p* = 0.083 (NPAC × OTS)
Chest-to-waist circumference ratio^*^	1.276 (±0.068)	1.249 (±0.062)	^*^(NPAC = 1.157 ± 0.069) *p* = 0.001 (overall), *p* = 0.0005 (NPAC × ATL) and *p* = 0.002 (NPAC × OTS)

* *These parameters were included because although p-value is above 0.05 between OTS-affected and healthy athletes, this is below 0.1, were significantly different between athletes (both groups) and sedentary (p < 0.05), and has potential physiopathological and clinical significance*.

*BW, Body Weight; POMS, Profile of mood states; BMR, Basal metabolic rate; T/E, Testoterone-to-estradiol ratio; OTS, Overtraining syndrome*.

**Figure 3:**
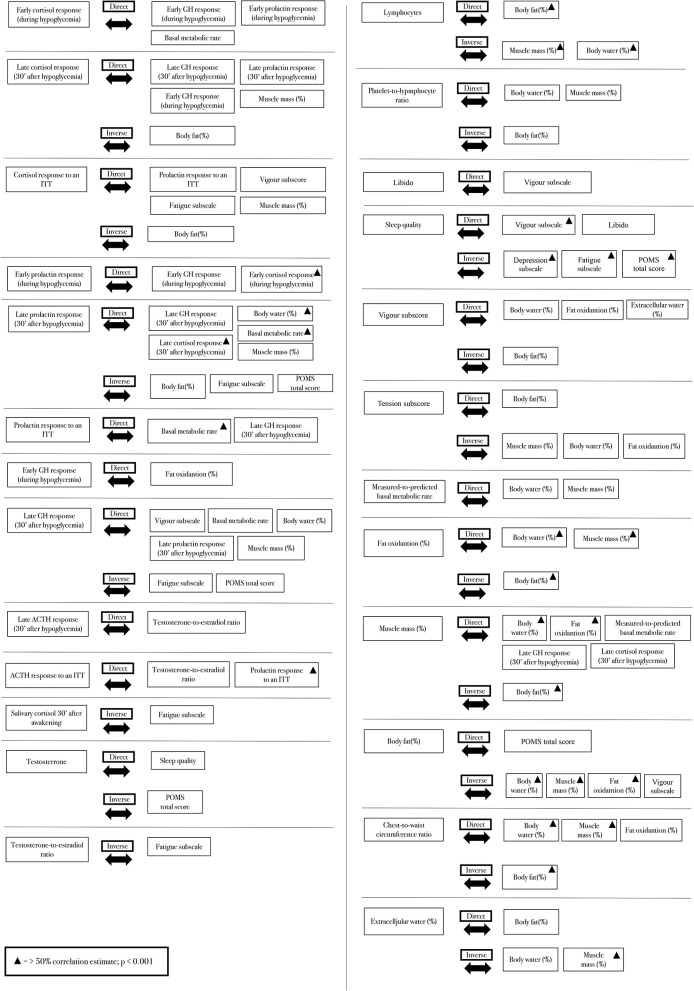
Strict correlations (>0.40) between clinical, hormonal, psychological, and metabolic parameters.

From the primary findings, further statistical analyses were employed, including the analyses for predictions and linear correlations, which are presented in Table 3 and Figure 3 [when >0.40 (*p* < 0.01)], respectively.

**Table 3:** Clinical or biochemical parameters as independent predictors of other parameters (multivariate linear regression analysis).

**Parameter**	***p* of the influence of the modifiable variables**	Level of influence by the modifiable variables^*^	**Parameters with significant correlations (and *p*-value)**	**Equation for the estimation of the parameter level in male athletes**
Total testosterone (ng/dL)	0.0415	8.4%	1. Fat mass (inverse) (*p* = 0.0415)	Testosterone (ng/dL) = 631.77–10.29 × (fat mass)
POMS anger subscore	0.0006	34.7%	1. Estradiol (inverse) (*p* = 0.008) 2. Presence of OTS (direct) (*p* = 0.003)	POMS anger subscore = 25.43–0.24 × (estradiol)−0.24(T:E ratio) + 6.97(if OTS)
Measured-to-predicted BMR (%)	0.026	10.9%	1. T:E ratio (direct) (*p* = 0.026)	BMR ratio (%) = 100.8 + 0.35(T:E ratio)
Fat oxidation (% of total BMR)	<0.0001 (together with extra-activities)	58.8%	1. Body water (%) (direct) (*p* = 0.001)	Fat oxidation = −66.96 + 2.30x(body water) + 0.51 × (T/E ratio)−4.99 × (extra activities)
Chest-to-waist circumference ratio	0.0003	37.8%	1. Visceral fat (inverse) (*p* <0.0001) 2. T:E ratio (inverse) (*p* = 0.038)	Ratio = 1.362–0.012 × (visceral fat)−0.02 × (T:E ratio)

* *Adjusted R-Square*.

*POMS, Profile of mood states; BMR, Basal metabolic rate; T/E, Testosterone-to-estradiol ratio; OTS, Overtraining syndrome*.

### Independent Predictors of Clinical, Metabolic, and Biochemical Biomarkers

None of the hormonal responses to the CST of the adrenal glands or the ITT predicted or was predicted by any of the other clinical or biochemical markers.

**Figure 4:**
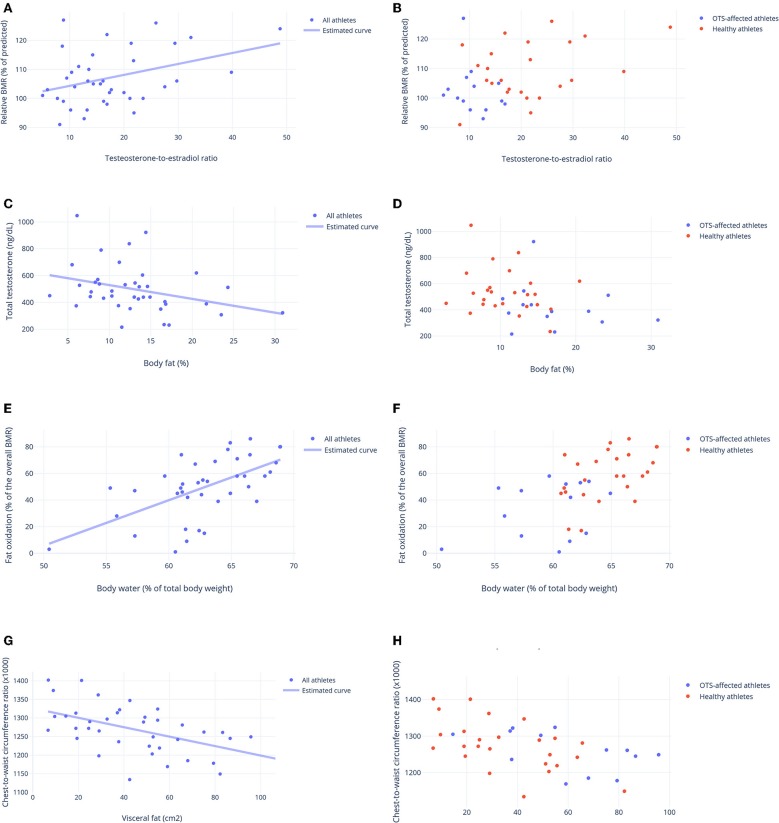
Biological predictors of other clinical, metabolic and biochemical parameters. **(A,B)** Testosterone-to-estradiol (T:E) ratio as a predictor of basal metabolic ratio (BMR) (relative to the predicted BMR, in %). Estimated equation: BMR ratio (%) = 100.8 + 0.35(T:E ratio)–*r* = 0.33. **(C,D)** Body fat (%) as a predictor of total testosterone (ng/dL) Estimated equation: Testosterone (ng/dL) = 631.77–10.29 × (fat mass-%)–*r* = 0.29. **(E,F)** Level of hydration (body water, in % of body weight) as a predictor of fat oxidation (% of total BMR) Estimated equation: Fat oxidation = −66.96 + 2.30 × (body water) + 0.51 × (T/E ratio)−4.99 × (extra activities)–*r* = 0.77. **(G,H)** Visceral fat (cm^2^) as a predictor of chest-to-waist circumference ratio. Estimated equation: Ratio = 1.362–0.012 × (visceral fat)−0.02 × (T:E ratio)–*r* = 0.62. **(A,C,E,G)** Estimated curve for all athletes, adjusted for OTS, when needed. **(B,D,F,H)** Results for athletes of OTS and healthy groups. Each point represents the result of one athlete.

Conversely, among the basal hormones, the testosterone-to-estradiol (T:E) ratio, identified in the EROS study as a better predictor of performance and overall status than total testosterone or estradiol alone (21), positively predicted the measured-to-predicted BMR ratio (Figures 4A,B), as well as the chest-to-waist circumference ratio. Total testosterone was inversely predicted by fat mass (Figures 4C,D), and estradiol inversely predicted anger, both fat mass and anger mood were also influenced by the presence of OTS (24).

Body water content positively predicted fat oxidation (Figures 4E,F) and chest-to-waist circumference was inversely predicted by visceral fat (in addition to the T:E ratio) (Figures 4G,H), which was also predicted by OTS (24). None of the other psychological parameters, muscle mass, other biochemical parameters, or SCR independently predicted or were predicted by any of the other factors evaluated in this study.

Markers not listed in Table 3 were not independent predictors of other clinical or biochemical behaviors.

### Linear Correlations

**Figure 5:**
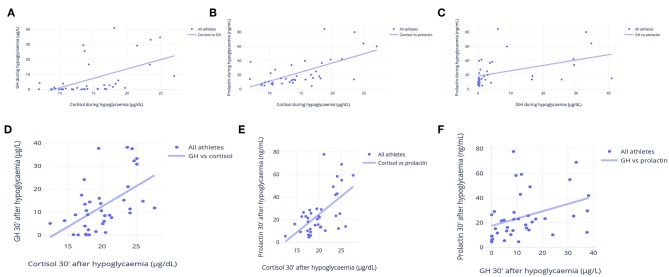
Correlations between cortisol, GH and prolactin responses to an insulin tolerance test (ITT). **(A)** Early cortisol (μg/dL) and GH (μg/L) responses to ITT (during hypoglycemia) (*R* = 0.72). **(B)** Early cortisol (μg/dL) and prolactin (ng/mL) responses to ITT (during hypoglycemia) (*R* = 0.80). **(C)** Early GH (μg/L) and prolactin (ng/mL) responses to ITT (during hypoglycemia) (*R* = 0.69). **(D)** Late cortisol (μg/dL) and GH (μg/L) responses to ITT (during hypoglycemia) (*R* = 0.77). **(E)** Late cortisol (μg/dL) and prolactin (ng/mL) responses to ITT (during hypoglycemia) (*R* = 0.76). **(F)** Late GH (μg/L) and prolactin (ng/mL) responses to ITT (during hypoglycemia) (*R* = 0.69). Each point represents the result of one athlete.

**Figure 6:**
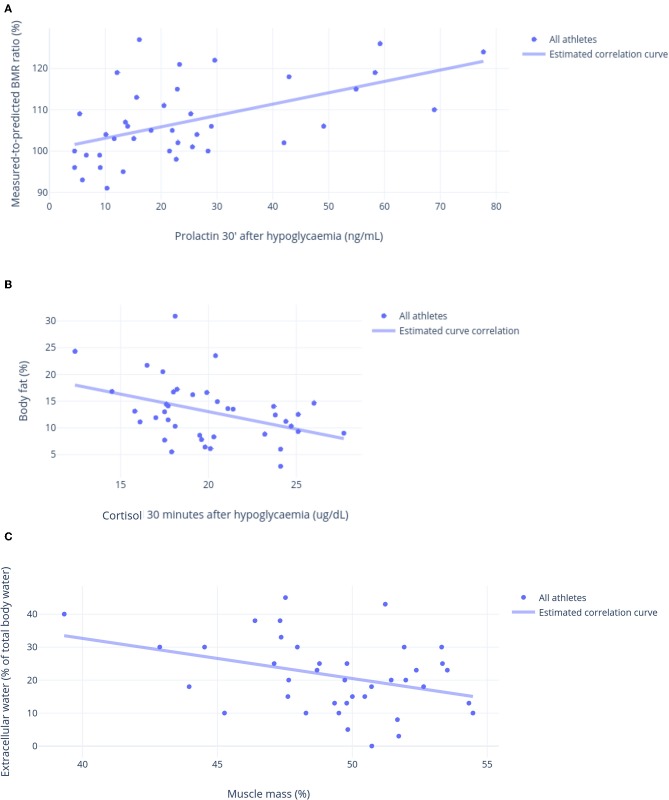
Other linear correlations. **(A)** Prolactin 30 min after hypoglycemia in response to an insulin tolerance test (ITT) (ng/mL) and relative basal metabolic ratio (% of predicted) (*R* = 0.71). **(B)** Cortisol 30 min after hypoglycemia in response to an insulin tolerance test (ITT) (μg/dL) and body fat (%) (*R* = 0.74). **(C)** Muscle mass (%) and extracellular water (% of body water) (*R* = 0.72).

Although none of the hormonal responses to the ITT predicted or was predicted by other parameters, both early and late growth hormone (GH), cortisol, and prolactin responses to the ITT were similar and had strong correlations (Figures 5A–F). These hormonal responses were positively correlated with vigor, fat oxidation, the predicted-to-measured BMR ratio, and muscle mass, and they were inversely correlated with fatigue and fat mass. The correlations between late prolactin response to the ITT (30 min after hypoglycemia) and relative BMR (% of predicted), and between late cortisol response to the ITT (30 min after hypoglycemia) and body fat are shown in Figures 6A,B, respectively.

Salivary cortisol 30 min after awakening and the T:E ratio were inversely correlated with fatigue, whereas total testosterone was inversely correlated with the Profile of Mood States (POMS) total score (a negative score indicates a better mood), and it was directly correlated with sleep quality. Immunologic parameters were correlated with body composition: body fat was directly correlated with lymphocytes and inversely correlated with the platelet-to-lymphocyte ratio, whereas body water and muscle mass were correlated with these variables in the opposite directions.

Libido and sleep quality were directly correlated with vigor, while sleep quality was directly correlated with libido and inversely correlated with depression, fatigue, and overall mood. Vigor was directly correlated with body water and fat oxidation, and inversely correlated with body fat; conversely, tension was inversely correlated with body water, muscle mass, and fat oxidation, and directly correlated with body fat.

The predicted-to-measured BMR ratio and fat oxidation had a stronger positive correlation with muscle mass and body water, whereas an inverse correlation was found between fat oxidation and body fat. Even though body fat and muscle mass were strongly and inversely correlated (*r* >-0.95), they were not always correlated in the opposite direction. In addition to fat oxidation, body water was the only parameter directly correlated with muscle mass, and inversely correlated with body fat. Although muscle mass was directly correlated with late GH and cortisol responses to the ITT, extracellular water (i.e., the presence of edema) and body fat were directly correlated with the POMS total score and inversely correlated with vigor.

Though the chest-to-waist circumference ratio was directly correlated with body water, muscle mass, and fat oxidation, and inversely correlated with body fat, extracellular water was correlated with these variables in the opposite directions (Figure 6C). The parameters that are not mentioned in this report failed to show strong correlations (>0.40).

Linear correlations not presented in Figure 3 were weaker than <0.40 (*p* > 0.01).

## Discussion

### The EROS Study and the Present Analysis

The EROS study elucidated some of the physiological adaptive changes that occur in healthy athletes and how these changes are disrupted in OTS (18–24), as this study addressed the major methodological issues in studies of healthy and OTS-affected athletes, using concurrent comparisons between sex-, age-, and BMI-matched healthy athletes and non-athletes, and simultaneous comparisons of a broad range of aspects within the same participants. The prior results for the two groups analyzed here (healthy and OTS-affected athletes) showed multiple clinical, metabolic, and biochemical conditioning processes in the healthy athletes and a loss of 59.1% of these conditioning processes in the OTS-affected athletes (23), which was referred to as “a mix of paradoxical deconditioning processes.”

The concomitant analysis of different biochemical, clinical, and metabolic aspects in the EROS study allowed us to explore the previously uninvestigated interactions, correlations, predictions, and synergistic actions between these parameters, using multivariate regression and other statistical techniques. Therefore, the EROS-CORRELATIONS study analyzed two major sorts of interactions among basal and stimulated hormonal, metabolic, immune, and muscular biomarkers, body composition and metabolism, and psychological patterns: independent predictors and linear correlations, as well as their mechanisms and outcomes.

Noteworthy, some markers previously hypothesized to be potential biomarkers for OTS yielded similar results between OTS and healthy athletes, including the testosterone-to-cortisol ratio (21) and the insulin growth factor-1 (IGF-1) (21). These markers also failed to demonstrate any sort of relationship with other markers, as shown in the raw statistical analysis (https://osf.io/bhpq9).

### Biochemical Responses as Predictors of Other Biochemical and Clinical Behaviors

Although fat mass is considered an independent suppressor of the GH response (28), this was not confirmed by our results, in that the body fat of male athletes was not important for the GH response. Although body fat did not have a negative effect on GH release, it reduced testosterone levels; however, the reduction was not due to increased aromatase activity, as the T:E ratio was not reduced by body fat or affected by any of the other markers. Conversely, the T:E ratio positively predicted the measured-to-predicted BMR ratio while neither testosterone nor estradiol had the same effect; the T:E ratio also predicted the chest-to-waist circumference ratio the measure of the torso's “V-shape.” Our findings underscore the importance of evaluating the ratios of different hormones for the prediction of metabolic outcomes. Although an increase in estradiol without a concurrent analysis of testosterone does not necessarily suggest either a beneficial or a harmful outcome, in this study, testosterone level did not predict any of the parameters, such as body metabolism or composition, without the simultaneous analysis of estradiol. These findings are consistent with studies suggesting a simultaneous increase in testosterone and estradiol has synergistic positive effects, which include metabolic parameters (29–33). Conversely, fat mass reduced testosterone levels, which can be justified by exposure of testosterone to a more intense aromatase enzyme under higher body fat (31, 32).

Although estradiol alone did not predict body metabolism or composition parameters, it independently predicted lower anger. Although estradiol receptors are widely distributed in the brain (29, 33, 34), their effects on mood in males are still unclear. Body water content was the only predictor of fat oxidation, which supports the premise that good hydration status, particularly within the cells, is a key requirement for proper fat oxidation, as water is part of this pathway (35–37), while dehydration slightly impaired fat burning.

### Linear Correlations

Strong inter-correlations were found among GH, prolactin, and cortisol for early and late responses to stimulation tests. Therefore, it was impossible to distinguish levels of responsiveness among the corticotropic, somatotropic, and lactotropic axes, as these hormones responded simultaneously and equally, which indicates a common, ubiquitous, and enhanced hypothalamic responsiveness in athletes, compared to sedentary controls. Although ACTH was not strictly correlated with the other hormones, its short half-life precluded drawing conclusions about its correlation with other hormones.

Late hormonal responses (30 min after hypoglycemia) were directly correlated with energy levels (higher vigor and lower fatigue levels), indicating a possible role for sustained hormonal release in response to stress in the prevention of burnout in chronically stressful situations. They were also correlated with increased muscle mass, lower body fat, and better hydration, without a distinction between the specific effects of each hormonal response (cortisol, GH, and prolactin). Acute cortisol and GH release promote fat oxidation and lipolysis, but chronic hypercortisolism may lead to the accumulation of visceral and central fat, even when it is mild.

Overall, the hormonal conditioning process that was found to occur in athletes, and identified by enhanced GH, prolactin and cortisol responses to exercise-independent stimulation tests, when compared to sex-, age-, and BMI-matched healthy sedentary controls, and adjusted for body composition, may be one of the underlying reasons for decreased extracellular water, decreased anger, fatigue, depression, confusion mood states, and indirect account for reduced fat, increased muscle, and better hydration (38). Indeed, under OTS, in which the optimization of the hormonal responses is lost (24), the concurrent benefits in response to intense exercise are also lost.

The inverse correlation between salivary cortisol 30 min after awakening and fatigue supported this parameter and the cortisol awakening response as markers of fatigue, as previously demonstrated by different studies (39–42). Indeed, cortisol awakening response (CAR), an indirect marker of cortisol 30 min after awakening, has been extensively used as a marker of fatigue (43–46). Nonetheless, they were not independent predictors of fatigue when analyzed using multivariate regression. Therefore, a lower increase in cortisol level between awakening and 30 min after awakening is unlikely to be the primary cause of fatigue, but rather a possible consequence of poor sleep quality leading to an impaired cortisol awakening response (47), with a blunted cortisol increase 30 min after awakening and a concurrent decrease in energy level (48–50), as well as other disruptions of the HPA axis (50). Thus, conclusions regarding adrenal function using these tools are inappropriate (51).

Testosterone level was directly correlated with sleep quality. Although this does not indicate a causal relationship, it supports the role of sleep quality in testosterone production because its' physiological peak also occurs in the early morning hours, which is affected by sleep quality (52–54).

The T:E ratio was also inversely correlated with fatigue, which implies that the ratio was a better predictor of energy level than either testosterone or estradiol alone. While estradiol alone was not linked to fatigue, its pathological increase, from an exacerbation of aromatase activity and consequent reduction of testosterone, may have increased fatigue, in accordance with the literature (32).

In the EROS study, although sleep duration did not have a role in psychological function, unlike previous studies, that strongly correlated sleep duration with mood states (49, 55, 56). Sleep quality was strongly correlated with better psychological outcomes, including lower depression and fatigue, and higher vigor, indicating that sleep quality led to a global improvement in mood, similarly to what has been unanimously observed (49, 55, 57–59). We speculate that sleep duration was not demonstrated to a major factor of mood states or any other characteristic in athletes because in higher quality sleep duration tends to play a less important role (60, 61).

Other moderate-to-strong linear correlations were identified. Body water was inversely correlated with lymphocyte and directly correlated with platelet-to-lymphocyte ratio. However, little data has been identified in the literature, specifically for these correlations (62–69), since immune function and hydration has been more assessed in athletes, in response to exercises (67–69). Specific absolute and lymphocytes subpopulation counting have been assessed in athletes and in patients at high cardiovascular risk, with indirect but inconsistent correlations between lymphocytes and hydration status (70–75). Platelet-to-lymphocyte ratio has been proposed as an inflammatory marker of cardiovascular risk, acute pancreatitis and sarcopenia (76–80), with some speculations that higher ratio could indicate better hydration status, similarly to the direct linear correlation that we found. This occurs perhaps due to a direct correlation between platelet count and total body water (65, 66, 81), although this associations remain controversial (64–68).

### Associations Between Improved Mood States and Body Composition and Metabolism

Vigor was directly correlated with better hydration, better fat oxidation, and less body fat, whereas libido was exclusively correlated with vigor, indicating improved overall body metabolism and composition, hydration, and fat oxidation. Since vigor has also been correlated with enhanced hormonal responses to stimulations and better sleep quality (24) in the present study, both of which have also been correlated with improved body metabolism and composition patterns, we hypothesize that vigor is an additional consequence of hormonal response and sleep quality, similarly to body composition and metabolism, and has no direct correlations of causal relationship with these parameters.

Conversely, while other moods were not significantly correlated with body metabolism or composition, tension had correlations in the opposite direction than those found for vigor. Oppositely to vigor, which unlikely leads to changes in hormonal responses, tension is likely the mood mostly correlated with disrupted hormonal responses (50). Once in our study tension was the mood state most strictly predicted by sleep quality, and that Impaired sleep independently leads to impaired muscle recovery (62), this may justify the correlations between vigor (positive) and tension (negative) and body muscle identified by the EROS study.

Also, although oxidative stress leads to muscle hypertrophy, chronic stress mediated by the HPA axis, leads to the opposite direction (82–85). Indeed, alterationsof the HPA axis disrupt the metabolism of the muscle tissue, toward a negative balance between protein generation and degradation, eventually leading to muscle mass loss (86–89). Conversely, impaired HPA axis also leads to concurrent independent body fat gain (84), as multiple mechanisms mediated by the HPA axis induce increase of fat cell size and induce a pro-inflammatory status irrespective of caloric balance, proportion of macronutrient intake, and sleeping patterns, since a post-receptor modification from cortisone to cortisol by enhanced activity of 11beta-hydroxysteroid dehydrogenase type 1(11beta-HSD1) (90, 91). In addition,disruption of the muscle metabolism, herein induced by altered cortisol regulation, has also demonstrated to have direct effects on the metabolism and accumulation of fat (92). Since the HPA axis is chronically and more severely enhanced by high tension levels, more than any other mood state, it would be expected, from this perspective, to find negative correlations between tension levels and body composition. Indeed, higher tension, which have been correlated with impaired hydration and fat oxidation, muscle catabolism, and increased body fat, are possibly due to the harmful metabolic effects of the chronic stimulation of the HPA axis (82–85), although direct effects of tension, depression, and anger on increased body fat has been observed (86).

In additional, overall mood states can hypothetically be indirect signals of hydration status, although direct relationships are unlikely.

### Hydration as a Key Characteristic for Athletes

Level of hydration is a key characteristic for the overall health status in humans, since water is a major participant of multiple reactions of metabolism and thermoregulation (37). Hydration, measured by the % of body water in relation to total body weight, was associated with multiple parameters (20, 22, 34), including both characteristics of body metabolism(the predicted-to-measured BMR ratio and fat oxidation), which is reasonable since water boosts fat metabolism (35, 36, 93, 94, 96) and overall metabolic rate (35, 94, 96–99), being considered as a potential thermogenic (35, 36, 93, 94, 96–98).

The need for a minimum amount of available water content for fat oxidation in catabolic states is conceivable in the context of a direct correlation between hydration and fat oxidation (35, 96). Conversely, the amount of body fat was inversely correlated with fat oxidation, which is expected, once more body fat may be a consequence of less fat utilization as source of energy, even under glycogen- and glucose-depleted circumstances, as observed by some studies (100, 101). Despite the previous reports on the correlation between body fat and fat oxidation, it is still unclear whether a larger fat mass resulted from reduced fat oxidation, or if a greater fat mass impaired fat oxidation through a non-classical inflammatory response that is enhanced in adipocytes when these are enlarged. Finally, since dehydration probably leads to lower fat oxidation, body water was expectedly found to be inversely correlated with body fat and the chest-to-waist circumference ratio.

Similar to the correlation between fat oxidation and body water, the measured-to-predicted BMR ratio was positively correlated with hydration status and muscle mass, which reinforces the extensive descriptions on the literature that body water content, hydration status, and muscle mass are the major components of the metabolic rate (94, 96–99, 102–104). Moreover, hydration has been correlated with vigor levels and other mood states at lesser extent, which is supported by the literature (105).

In regards with the location of the water—whether intra- or extracellular—extracellular water accounts for ~40% of total body water (106), which was similar to that observed in the healthy sedentary of the EROS study. However, in healthy athletes extracellular water was shown to be reduced, possibly as a mechanism of facilitation for the optimization of intracellular metabolic pathways. Among which most reactions require water to occur. In the EROS study, in the groups of athletes ~80% of the water was located intracellularly, which can be considered as an improvement of the water balance for metabolic purposes (107). Conversely, fat mass is inversely correlated with relative and absolute intracellular water, and consequently lower body water. Some authors speculate that this is possibly due to the fact that enlarged fat cells tend to become more hydrophobic and to contain less water. However, this is still a hypothesis to be further demonstrated.

We observed an inverse correlation between hydration status and extracellular water, i.e., better hydration was correlated with less edema, indicating that the amount of water consumed may redirect water toward inside cells (the preferred space), rather than interstitially (third space; extracellular water). However, external factors such as disturbed hormonal secretion, excessive sodium intake, and hypercaloric diets may lead to excessive extracellular water, which becomes the primary cause of dehydration: resulted from shifts in water compartment redistribution (106, 108). Indeed, dehydration may not only be a result from redistribution of water, but overall low water content can also induce further dehydration by its accumulation in the extracellular water, as a mechanism of protection against further water wasting, secondarily to vasopressin (ADH) and renin-angiotensin-aldosterone-system (RAAS) metabolism (109). In addition, interactions between water content, the RAAS, the HPA axis, the direct aldosterone actions and their relationship with chronic stress have been reported (105, 109).

In the EROS study, the major influences that drove the water destination were the metabolic environment, sleep quality (worse sleep leads to worse hydration and increased edema), the amount of muscle mass, eating patterns, and mood. Muscle mass was positively correlated with body water and it may have indirectly prevented edema, while body fat may have had the opposite effect, similarly to the observed in the literature (86, 98).

### Implications of the Findings

The use of additional statistical techniques in this study facilitated the identification of independent predictors of linear correlations among clinical, metabolic, and biochemical parameters and other parameters, which has improved our understanding of hormonal and metabolic behaviors, and their multiple interactions and influences in athletes. They also yielded information to speculate on new potential markers and new understandings of current markers.

The summary of the findings and their possible implications are presented in Table 3. Hydration status, and, to a lesser extent, muscle mass, were the two major determinants of metabolic rate and fat oxidation (35, 93–99, 102–104). These results support the importance of adequate water intake and maintaining and building lean muscle for adequate metabolism and fat oxidation. The body fat effect on GH release was attenuated in athletes, while its effect on testosterone was maintained, suggesting that athletes with excessive body fat might not benefit from some of effects of exercise, which is a reason for sports professionals to have a good body shape.

Since the hormonal responses to an ITT were strictly correlated, i.e., the level of increase of GH, cortisol, ACTH, and prolactin was similar within each athlete (Figure 5), the level of hypothalamic responsiveness to stimulation seemed to be diffuse, rather than specific for certain axes. Energy levels were strongly correlated with hormonal status, including a prolonged optimization of hormonal responses and a better cortisol response to awakening, although a causal relationship was undefined. In addition to energy levels, better hormonal responses were correlated with better body composition, of which the causal relationship remains uncertain.

Sleep quality seemed to be the most important factor in mood, rather than any other factor, such as hormonal levels or eating patterns, which has been demonstrated to play an essential role on overall cognitive and psychological functions (REF), in accordance with our findings. The level of hydration was inversely correlated with edema and better hydration was linked to less edema, depending on the location of the water in the body, regulated by external factors rather than the amount of water intake.

### Testosterone, Estradiol, and Testosterone-To-Estradiol (T:E) Ratio

Testosterone, estradiol, and their ratio (T:E ratio) had different roles and influences. While testosterone was inversely related to body fat, positively linked to sleep quality, and indirectly linked to improved psychological outcomes, alone, it did not predict any of the parameters. Conversely, estradiol unexpectedly predicted anger, because of its actions on the male brains of the athletes. The T:E ratio had the most important roles in body metabolism and composition, and was linked to energy level. Hence, the balance between testosterone and estradiol might be more important than either testosterone or estradiol alone. This is extensively supported by the literature, since the T:E ratio predicts multiple outcomes, including cardiovascular changes (29, 30), while many of the neuroprotective and psychological effects of estradiol in males are mediated by testosterone (32–34), which requires a balance between these hormones to obtain the health benefits. The unaltered balance is more precisely assessed by the T:E ratio, rather than each hormone alone.

The T:E ratio is a significant ratio that has a promising role in the evaluation of athletes. It was found to be a better predictor of metabolic and psychological parameters than either testosterone or estradiol alone, supporting the hypothesis that it is a potential novel parameter. We based this hypothesis on a new understanding of the role of estradiol in males, and identified two types of estradiol increase: (1) a physiological increase, secondary to an increase in testosterone, when high testosterone levels are maintained and (2) a pathological increase, caused by an aberrant exacerbation of aromatase activity, leading to a decrease in testosterone. We found the most appropriate way to differentiate these situations objectively was to examine the T:E ratio. This ratio indicates whether an increase in estradiol is followed by an increase in testosterone; it should remain unaffected in the case of a physiological increase. These data were supported by a recent study showing that increased estradiol benefitted males in terms of increasing their libido, muscle mass, and bone mass, and reducing their fat mass, but only when accompanied by increased testosterone levels (26–28), which occurs when increased estradiol is actually desirable and the T:E ratio is unaffected (29). Conversely, the estradiol increase that we identified as a marker of OTS was due to a pathological conversion from testosterone, indicated by a substantial decrease in the T:E ratio, which was most likely a response to an anti-anabolic environment. For practical purposes, the T:E ratio should be above 13.7:1 (21).

### Summary of the Findings

The EROS-CORRELATIONS study demonstrated that testosterone was predicted by fat mass, estradiol predicted anger, and the T:E ratio predicted the measured-to-predicted BMR ratio and chest-to-waist circumference, while hydration status predicted fat oxidation. Early and late somatotropic, corticotropic, and lactotropic responses were strong and strongly correlated, showing a diffuse hypothalamic rather than axis-specific response to stimulation. Late hormonal responses to stimulations, increased cortisol after awakening, and the T:E ratio was correlated with energy level. Sleep quality was the major factor correlated with most of the study's psychological measures, while fat oxidation, hydration, muscle mass, and body fat were highly inter-correlated, and edema was inversely correlated with hydration and muscle mass, and directly correlated with fat mass. The most remarkable findings are described in Table 4.

**Table 4:** Most remarkable findings of the EROS-CORRELATIONS study.

**Parameter**	**Markers**	**Potential implication(s)**
**TESTOSTERONE, ESTRADIOL AND T:E RATIO**		
Total testosterone	(1) Decreased body fat (P) (2) Better sleep quality (C)	1. Testosterone is blunted by body fat 2. Better sleep quality may boost testosterone production
Estradiol	(1) Lower anger levels (P)	1. Estradiol actions in the male brain improve anger levels
Testosterone-to-estradiol ratio	(1) Increased measured-to-predicted basal metabolic rate (P) (2) Increased chest-to-waist circumference ratio (P) (3) Lower fatigue levels (C)	1. The ratio between testosterone and estradiol is more important than testosterone or estradiol alone for body metabolism and composition
**HORMONAL FUNCTIONAL TESTS**		
GH, prolactin, and cortisol responses to an insulin tolerance test	(1) Positive (direct) inter-correlations between GH, prolactin, and cortisol in early responses (C) (2) Positive (direct) inter-correlations between GH, prolactin, and cortisol in late responses (C) (3) Lower body fat (C) (4) Higher fat oxidation (C) (5) Higher muscle mass (C) (6) Better hydration (C) (7) Lower fatigue levels (C)	1. Hypothalamic responsiveness to stimulations does not discriminate between different axes 2. Although causality is not confirmed, better hormonal responses are at least linked to more energy and to better body composition
**SOCIAL AND PSYCHOLOGICAL ASPECTS**		
Sleep quality	(1) Improved overall mood states (C) (2) Lower depression levels (C) (3) Less fatigue levels (C) (4) Higher vigor levels (C)	1. Sleep quality may be more important than hormonal levels or eating patterns for the psychological status of the athletes
Libido	(1) Higher vigor levels (C)	
Vigor	(1) Lower body fat (C) (2) Higher fat oxidation (C) (3) Better hydration (C) (4) Higher extracellular water (C)	1. Vigor is an indirect marker of less body fat, better fat oxidation, and lower edema
Tension	(1) Higher body fat (C) (2) Lower fat oxidation (C) (3) Worse hydration (C) (4) Lower muscle mass (C)	1. Tension is an indirect marker of lower muscle mass, increase of body fat, impaired fat oxidation, and less hydration
**BODY METABOLISM AN COMPOSITION**		
Measured-to-predicted basal metabolic ratio	(1) Higher testosterone-to-estradiol ratio (P) (2) Better hydration (P) (3) Higher muscle mass (C)	1. The balance between testosterone and estradiol, more than any hormone alone, is the major predictor of metabolic rate in male athletes 2. Together with the T:E ratio, body water and muscle mass are the two major contributors of the metabolic rate, which means that a minimum content of intracellular water is necessary for a proper metabolism
Fat oxidation	(1) Better hydration (C) (2) Higher muscle mass (C) (3) Lower body fat (C)	3. Body water and muscle mass play the most important roles for fat oxidation, the first as part of the pathway for fat oxidation, and the second as a possible signaller for the selective fat catabolism, over protein catabolism 4. Body fat and fat oxidation are inversely correlated; however, whether fat-induced inflammation leads to reduced fat oxidation, or higher body fat is a result from reduced fat oxidation, is unknown
Chest-to-waist circumference ratio	(1) Higher testosterone-to-estradiol ratio (P) (2) Lower visceral fat (P) (3) Higher muscle mass (C) (4) Higher fat oxidation (C) (5) Better hydration (C) (6) Lower body fat (C)	1. Similarly to other metabolic parameters, the T:E ratio is the most important direct predictor of the W:C ratio, leading to the popular “V-shape,” highly correlated with an androgen phenotype. 2. Once body water is the intracellular water, mostly located within miocytes, rather than adipocytes, this contributes for a higher W/C ratio 3. Muscle mass and body fat are expectedly directly and inversely correlated with W/C ratio, respectively
Muscle mass	(1) Late GH response to stimulation (C) (2) Late cortisol response to stimulation (C) (3) Better hydration (C) (4) Higher fat oxidation (C) (5) Lower body fat (C)	1. Although the muscle mass is not the lean mass, i.e., the water within muscles are not accounted, the presence of body water helps provide a muscle anabolic environment, and predicts fat oxidation. 2. Late hormonal responses, although correlated with muscle mass, are probably two consequences of a same common factor.
Fat mass	(1) Improved overall mood states (C) (2) Higher vigor levels (C) (3) Decreased hydration (C) (4) Lower muscle mass (C) (5) Decreased fat oxidation (C)	1. Worse psychological moods may be indicators of less healthier environment, that naturally tends to save fat storage and catabolize muscle mass. 2. All correlated body composition parameters are accordingly.
Extracellular water (= edema)	(1) Worse hydration (C) (2) Lower muscle mass (C) (3) Increased fat mass (C)	1. The more proper hydration, the less edema; however, what determines the destination of the ingested water is the metabolic environment, not the amount of water intake 2. Fat mass, likely through inflammatory processes, may induce edema, although we did not find prediction relationship.

*P, Prediction; C, Correlation; T:E, Testosterone-to-estradiol; W:C, Chest-to-waist circumference*.

## Limitations

The findings of the present study are valid only for male athletes that practice both endurance and strength sports, as basal hormone levels and responses to stimulations are highly sex-specific and may be sport-specific. Hence, different arms of the EROS study focused on purely strength, purely endurance, purely explosive, purely stop-and-go, and mixed sports, conducted with male and female participants, should provide data that are more specific. Given the unexpected findings regarding several hormones and other biochemical markers, we suggest additional parameters for further studies, including luteinizing hormone, follicle-stimulating hormone, sex hormone-binding globulin the tumor necrosis factor-alpha, interleukin-1 beta, lactate dehydrogenase (LDH), free thyroxin-4, and cortisol binding globulin. Longer stimulation tests, including thyrotrophic and gonadotrophic responses (given the unexpected response of the lactotropic axis), and an examination of the associations between exercise-dependent and exercise-independent tests should also be examined. Also, the estradiol levels in males may lose absolute precise using chemoiluminescence, compared to liquid chromatography mass spectrometry/tandem mass (LC/MS-MS/MS). However, the relative precision is highly accurate, which allows the in-between (pairwise) group comparisons as fully satisfactory (110–115).

## Final Discussion

We found multiple correlations and predictions between clinical, hormonal, biochemical markers, that occurred as a web of influences, as multiple and multi-directional chain-reactions, that allowed us speculate on several new mechanisms to occur in response to sports. The identification of a complex web of interactions among many different aspects allowed us to hypothesize that sports performance results from a combination of hormonal, energy, and water availability, and psychological and muscular status The predictions, correlations, and interactions revealed in the present study show that further studies should not evaluate each aspect separately, as this is unlikely to provide answers to important questions.

## Data Availability Statement

The datasets generated for this study are available on request to the corresponding author.

## Ethics Statement

The studies involving human participants were reviewed and approved by Ethical committee of the Federal University of São Paulo (Approval Number: 1093965). The patients/participants provided their written informed consent to participate in this study.

## Author Contributions

FC and CK developed the central idea of the present manuscript. FC performed the tests of the EROS study, compilated the data, analyzed the results, and participated in the discussions. CK actively participated in the discussion, supervised and reviewed the results, helped with the final version of the manuscript, and gave the last word before the submission. All authors have read and approved the manuscript.

### Conflict of Interest

The authors declare that the research was conducted in the absence of any commercial or financial relationships that could be construed as a potential conflict of interest. The handling editor is currently co-organizing a Research Topic with one of the authors FC and CK, and confirms the absence of any other collaboration.
